# The mental health care model in Brazil: analyses of the funding, governance processes, and mechanisms of assessment

**DOI:** 10.1590/S1518-8787.2017051006059

**Published:** 2017-02-21

**Authors:** Thiago Lavras Trapé, Rosana Onocko Campos

**Affiliations:** I Faculdade São Leopoldo Mandic. Campinas, SP, Brasil; II Departamento de Medicina Preventiva e Social. Universidade de São Paulo. São Paulo, SP, Brasil; III Departamento de Saúde Coletiva. Universidade Estadual de Campinas. Campinas, SP, Brasil

**Keywords:** Mental Health Services, organization & administration, Mental Health Services, economics, Health Services Administration, Health Personnel Management, Health Services Evaluation

## Abstract

**OBJECTIVE:**

This study aims to analyze the current status of the mental health care model of the Brazilian Unified Health System, according to its funding, governance processes, and mechanisms of assessment.

**METHODS:**

We have carried out a documentary analysis of the ordinances, technical reports, conference reports, normative resolutions, and decrees from 2009 to 2014.

**RESULTS:**

This is a time of consolidation of the psychosocial model, with expansion of the health care network and inversion of the funding for community services with a strong emphasis on the area of crack cocaine and other drugs. Mental health is an underfunded area within the chronically underfunded Brazilian Unified Health System. The governance model constrains the progress of essential services, which creates the need for the incorporation of a process of regionalization of the management. The mechanisms of assessment are not incorporated into the health policy in the bureaucratic field.

**CONCLUSIONS:**

There is a need to expand the global funding of the area of health, specifically mental health, which has been shown to be a successful policy. The current focus of the policy seems to be archaic in relation to the precepts of the psychosocial model. Mechanisms of assessment need to be expanded.

## RESUMO

**OBJETIVO:**

Analisar o estágio atual do modelo de atenção à saúde mental do Sistema Único de Saúde, segundo seu financiamento, processos de governança e mecanismos de avaliação.

**MÉTODOS:**

Foi realizada uma análise documental de portarias, informes técnicos, relatórios de conferência, resoluções e decretos de 2009 a 2014.

**RESULTADOS:**

Trata-se de um momento de consolidação do modelo psicossocial, com ampliação da rede assistencial, inversão de financiamento para serviços comunitários com forte ênfase na área de crack e outras drogas. A saúde mental é uma área subfinanciada dentro do subfinanciamento crônico do Sistema Único de Saúde. O modelo de governança constrange o avanço de serviços essenciais, havendo a necessidade da incorporação de um processo de regionalização da gestão. Os mecanismos avaliativos no campo burocrático se mostram pouco incorporados à política de saúde.

**CONCLUSÕES:**

É necessário ampliar o financiamento global da saúde e específico da saúde mental, que vem se constituindo como uma política exitosa. O foco atual da política se mostra anacrônico aos preceitos do modelo psicossocial. Aponta-se a necessidade de ampliação de mecanismos avaliativos.

## INTRODUCTION

A “model” is understood not as something to be followed, but as a reflection of the reality of an organization^1^. It encompasses techniques, technologies, concepts, and theories in a certain social, political, and historical moment^2^, being a type of organization that tries to answer to a given demand, using a certain rationality which guides the practices.

The consolidation of health care models happens in complementary contexts: professional (new clinical technologies, training process, etc.), institutional (creation of new services, management practices, etc.), and systemic (legal framework, governance, funding, and assessment and control methods) practices. The relationship between them is plastic. Organizational changes at the systemic level influence the daily life of the services, while the reality of the units imposes changes to the hegemonic model, either from the consensus of the players involved or from the ancestry of interest groups in the political decision-making process.

The Brazilian Unified Health System (SUS) has created responsibilities in the management and funding of the health for the three federated levels (Country, State and Cities). This organization increases the complexity of the processes of construction and dissemination of the health policy as a whole and gives a strategic role to the city in the implementation of health policies^3^. The process of decentralization was important in the last two decades to consolidate new forms of management, offer new services, and increase social participation. In this scenario, new experiences have appeared in the field of mental health, anchored in the creation of community services and closing of hospital beds. It was a reality that is still concentrated on cities with greater management capacity that validated the new model, helping boost the systemic changes^4^.

In the early 1990s, the Coordination of Mental Health of the Ministry of Health promoted changes in the funding that regulated and typified the still incipient *Centros de Atenção Psicossocial* (CAPS – Psychosocial Care Centers) and *Núcleos de Apoio Psicossocial* (NAPS – Psychosocial Support Centers), supporting their multiplication^5^. At that stage, it was clear that the increased funding and direct destination to specific mental health services were key elements to start the transition of the model, being a strategy of induction. As a result of this policy, there was a change in the number of admissions in relation to the offering of new community services, with a decrease of 12.8% compared to the growth of 99% of the CAPS in the period from 1997 to 2001^6^.

The creation or removal of a health care model also depends on the answers that it gives from its objectives. The field of health assessment was developed being related to the process of democratization of the country and the increased investment in government health programs. Furtado and Vieira-of-Silva^7^ separate the assessment into two design spaces: bureaucratic and academic. On the one hand, when performed by units linked to Government spaces, the assessment tends to have lower plasticity and greater heteronomy; however, it has greater applicability in the reality assessed. On the other hand, the academic groups are characterized by having methodological variety and autonomy, but their results are less likely to be used.

In the case of mental health, the assessment tools were used as technical means to report a model that was, so far, weak. We highlight, in the bureaucratic field, the *Programa Nacional de Avaliação do Sistema Hospitalar/Psiquiatria* (PNASH – National Program of Assessment of the Hospital/Psychiatry System), which allowed the closing of many beds and helped in the qualification of the hospital offer^6^.

After twelve years in Congress, Law 10,216^a^, known as the Law of Psychiatric Reform and its subsequent Ordinances, imposed a legal value to the changes that were occurring in Brazil and allowed them to grow from the funding and creation of new services. Since its enactment, federal funding in mental health had a real growth^8^, mainly in the areas focused on the creation of new community services, in addition to a gradual reduction of funding for hospital beds^b^. At the end of the last decade, we had a reality of more services replacing the psychiatric hospital, with significant reduction in the number of beds. In 2006, the value allocated to community services was greater than the expenditure with hospital beds, following international standards.

Thornicroft and Tansela^9^ have reviewed more than 3,000 articles from 1980 to 2003 and have found that the best results in mental health care models are in “balanced care models”, with expansion of community services and hospitalization, preferably in general hospitals. The authors also indicate the differences in the models in relation to funding, separating the countries with high investment in mental health and those that invest little and restrict the care services^10^. The focus of the funding combined with efforts to monitor it, based on the assumptions discussed, is strategic for the improvement of the mental health care, even in countries with poor care resources^11^.

In Brazil, regarding the assessment processes, the growth of studies on the field of mental health is notorious, focused on different forms of services that make up the health care network^12^ ^,^ ^13^. However, even in the face of the growth in the number of assessment studies on mental health, the scenario is still little explored and the studies have not influenced the macro-political context^14^. Health funding^15^, specifically in mental health, is insufficient and the governance process, with great autonomy of cities, has been showing signs of being saturated and with explicit need to be changed^16^.

This study aimed to analyze the current stage of the Brazilian mental health care policy by analyzing its model and its macro-structural components, related to the governance and funding process and the mechanisms of assessment.

## METHODS

This is a qualitative and evaluative study about the official regulations of the Brazilian mental health care system. We have carried out a review and analysis^17^ of the guiding documents of the current mental health policy at the Federal context, which consolidate the Brazilian psychosocial model, from 2009 to 2014, including Laws, Conferences, Decrees, Ordinances, and Technical Reports of the Ministry of Health (MS). We have incorporated documents from before the study period, as they still structure the Policy and are a reference for current documents.

Altogether, we have analyzed five Ordinances, two Decrees, two Conference Reports, one Resolution, one Technical Report, and one electronic database. The material has been organized and analyzed from the definition of the model and the trinomial Funding-Governance-Assessment, used as axes for understanding the current stage of the mental health policy (Table).

**Table:** Documents analyzed.

•Decreto nº 7.179, de 20 de maio de 2010.
•Decreto nº 7.508, de 28 de junho de 2011.
•Saúde Mental em Dados – 10, ano VII, nº 10. Informativo eletrônico. Brasília: março de 2012 (acesso em 3/10/2014).
•Observatório, Crack é possível vencer.
•Portaria nº 1.654, de 19 de julho de 2011.
•Portaria nº 130, de 26 de janeiro de 2012.
•Portaria nº 131, de 26 de janeiro de 2012.
•Portaria nº 3.088, de 23 de dezembro de 2011.
•Portaria nº 4.279, de 30 de dezembro de 2010.
•Relatório final da IV Conferência Nacional de Saúde Mental – Intersetorial. Ministério da Saúde/Comissão Relatora da IV Conferência Nacional de Saúde Mental – Intersetorial, Brasil.
•Relatório final da XIV Conferência Nacional de Saúde. Ministério da Saúde (2011).
•Resolução nº 448, de 6 de outubro de 2011.

## RESULTS AND DISCUSSION

Recently, the MS published Decree 7,508^c^, which defines the components required to make a Health Region, with care points arranged in vertical structures, including the psychosocial care. The psychosocial care is the only specialized area that is contemplated in this text and still is a way to enter the System.

As a development of this Decree, the MS published a number of Ordinances establishing “Thematic Networks”, considered a priority for the reorganization of the health care model. Ordinance 3,088^d^ created the *Rede de Atenção Psicossocial* (RAPS – Network of Psychosocial Care) and defined the model of mental health care based on community care, social participation, and emphasis on the care to the user of crack cocaine, alcohol, and other drugs, with a regional management model. The objectives of these Guidelines include the increased access of users to the care and the focus on users of psychoactive substances.

The existing care points are organized by vertical axes: primary health care, specialized psychosocial care, urgent and emergency care, transient residential care, hospital care, strategies of deinstitutionalization, and psychosocial rehabilitation. It is clear in this component the diversification of the units and the congruence of the official policy with the changes produced in the care model in recent decades.

However, the efforts recommended by the care points suggest some anachronisms. In the basic health units, only prevention and health promotion efforts are included, preferably shared with other care points. However, in some Brazilian cities, basic health units have fixed mental health professionals, who can, if well-coordinated, offer care efforts with good results^18^. In this perspective, the Ordinance defines the *Núcleo de Apoio à Saúde da Família* (NASF – Family Health Support Center) as the unit responsible for developing these efforts and does not allow any other type of arrangement possible for primary care.

In the urgency and emergency care points, important for the consolidation of the model, the type III CAPS, which have been shown to be a strategic space of reception and management in crisis situations^19^, are not even mentioned. In this same component, the primary care is considered a care point.

In the content of the RAPS, it is clear the intention to change the operating logic of hospitalizations. It defines “referral hospitals” as a care point, makes exceptions for the compliance to Law 10,216^a^, and tries to stipulate the length of stay as short stay. It marks the intention of keeping the progressive closing of beds in psychiatric hospitals, defining that they can only co-exist with the RAPS in places where the implementation process is not complete. The CAPS are a central part in the organizational proposal of the RAPS. In all the components analyzed, it appears as a “partner” needed for projects, in addition to being the organizer of the health care flow to the hospital level.

Another mark of the current model is the constant reference to the area of crack cocaine, alcohol, and other drugs. The Federal Government released in 2010 the Decree establishing the *Plano Integrado de Enfrentamento ao Crack e outras Drogas* (Integrated Plan for Combating Crack and Other Drugs), which is conducted by the Ministry of Justice. A series of assignments was created for the sectors involved with the *Programa Crack é Possível Vencer* (PCPV – Program Crack: it is Possible to Win)^e^. The Ministry of Health is part of the management committee and has instituted a Plan to expand the health care network, increase hospital beds, and carry out preventive and educational works. The National Health Council showed concern regarding the development of the Program and, from Resolution 448^f^, reaffirmed the principles of the psychosocial model.

### Governance

The current mental health policy is concerned in advancing the regional pacts, making references to the health regions in the organizational process as part of a series of changes in the legal scope of the SUS. There are specific guidelines so that the planning of the RAPS takes place with the Regional Management Committees (CGR), which are spaces with representatives from the cities of a particular health region and the State. This guideline follows the governance model referred to in Ordinance 4,279^g^, which establishes the guidelines for the establishment of Health Care Networks.

The governance process of the SUS differs from a process of vertical governance, since it requires the effective participation of various governmental and non-governmental players in the debate and settlements of policy directions. However, it still requires a legal instrument that intermediates these relationships in order to establish the collective choices and define the role of each entity in the decision-making process and policy building^20^.

Decree 7,508 establishes the *Contrato Organizativo de Ação Pública da Saúde* (COAP – Organizational Agreement of Public Health Action) as an instrument of regional agreement, from which roles and responsibilities should be defined for each entity within a regional strategy. The Decree also combines part of the funding to the health regions. The need for the area of mental health to have this organizational change is justified by the saturation in the expansion of essential services in the current model (until 2011, 43% of type III CAPS were concentrated on the State of São Paulo)^18^. In short, the expansion of the type III CAPS network depends on two points: greater financial incentive for more services at the expense of less complex units and the creation of health regions, which can respond for more than 5,200 cities with less than 200,000 inhabitants, increasing its management and supply capacity.

The response capacity of a CAPS of regional level is under debate. There is experience of two type III CAPS managed by the state of São Paulo that participated in an assessment process together with 95% of all type III CAPS of the State^21^. The results presented by them did not highlight positive or negative aspects that differentiated them from the other ones (of city management).

The important aspect in this debate is the ability to think a regional management of mental health services, as we come from a tradition of construction that has its logic based on cities. In addition, regionalization imposes a new placement of the States in the participation of health policies, taking a central role in the governance spaces and management processes.

### Funding

The focus of the funding in the area of mental health follows the trend of countries with consolidated health systems^f^ ^,^ ^h^, which prioritize community health services, being endorsed by the instances of social control. However, the World Health Organization advises that investment in mental health should be approximately 5% of the health budget, as a result of its prevalence and the impact in the health care. The Brazilian Government spends only 2.3% of the total health budget in actions for mental health. This value increases slightly if we consider mental health in primary care and the Family Health Support Centers (NASF), which are not referred to in the studies analyzed^8^. This datum shows that the real growth does not reflect the percentage, which has not increased and needs to be reviewed for the consolidation of the model. Mental health is underfunded within an underfunded system.

The last National Conference on Mental Health^i^ shows a concern to ensure adequate funding for the provision and maintenance of substitute services, increasingly proposing federal control of the money applied. Although the responsibility for the funding is interfederative, the impact of the federal underfunding has given to the cities a strangulation of the public expenditure. There are no reliable sources that define the percentage of the expenditure of States and cities on specific programs and services for mental health, a fact that is worthy of research studies and assessments.

Inserted in this context, the PCPV has no budget allocation to fulfill its goals, according to its article 6^e^. The funds allocated to the cities, in the “fund-to-fund” model, consist of three blocks: management of the SUS, medium and high complexity, and investment, pertaining specifically to the construction of new units of the Program.

The values for the payment of the services vary according to their complexity^d^. Recently, the Ministry of Health has increased the value allocated to the type III CAPS^j^ in little more than 30% of the value of the monthly cost. This change represents an attempt to impute a necessary change to the care model, increasing the still scarce type III CAPS in the country; however, we can see a prioritization of the services related to PCPV.

The type III CAPS for AD (alcohol and other drugs), advocated by the program, receive 25% more than the same service for any other typology, therefore inducing the opening of fifty-nine units since the beginning of the Program. Ordinance 131^k^ regulates the funding for “therapeutic communities” using a series of policies concerning the structure, staff, and ways to join them. We highlight that this care point is already one of the biggest and most expensive parts of the Program, working against Law 10,216^a^, which highlights inconsistencies in the regulatory standards.

It is clear the need for studies on the assessment and cost-effectiveness of the services offered. Furthermore, the prioritization regarding the investment block and the increased funding indicates the induction of a model and reflects the current focus of the policy, which has the PCPV as the central program and the therapeutic communities as protagonists.

### Assessment

The final reports of the Conference on Health and Mental Health^e^ have been reflecting the concern to monitor and assess the quality of the services. Similar situation can be seen with the Ordinance of the RAPS^d^, which defines the assessment of services using indicators as one of its objectives and it also says that a shared assessment process should be carried out in the several care points together with States and the CGR.

Gonçalves et al.^8^ have already pointed out the need to expand the assessments that could show the power of the psychosocial model, without the presence of the false inference that the reduction of psychiatric beds in hospitals is indicative of a lack in health care. In the academic field, there is a vast production of assessments of services and technologies in the area of mental health, mainly from the first decade of the 2000s, encouraged by research notices such as the Research Program for the SUS.

The research studies that sought to assess the current model with participatory methodologies and State scope have not been incorporated into the assessment processes of government entities. An example is the study of Furtado et al.^21^, who have assessed 95% of the type III CAPS of the state of São Paulo (48% of the total in Brazil) and have defined a range of indicators that could encourage the creation of mechanisms for assessment and control in different aspects of the policy. We can say that there is already a tradition of using methods of assessment in the universe of mental health services, which are little used in the context of broader public policies.

The assessments presented annually by the Technical Department of Mental Health (Mental Health in Data) make up a document that shows some points of the model related to the expansion and provision of more services, reduction of beds, and amount financed. It is an institutional document, which aims to show the progress of the policy, bringing data that are important and convenient to the technical area of the Ministry of Health, without progress in the analyses.

We do not currently have a space of assessment of national scope that can analyze the policy in its macro-structural context. This can be seen by the low incorporation of references related to the mental health policy in the two major initiatives to assess the network of health services, the *Programa Nacional de Melhoria do Acesso e da Qualidade da Atenção Básica* (PMAQ – National Program for Improving the Access and Quality of Primary Care) and the *Índice de Desempenho do SUS* (IDSUS – Performance Index of the SUS). When it comes to academic research, most studies have a restricted range, related to production capacity and interests of research groups, in addition to being rare and, often, having mixed results^14^.

The assessment concerning the PCPV, according to the Ordinances, is the responsibility of the State and city health departments, with support from the Ministry of Health. We have to ask if, without the existence of a national policy of assessment and centralized control, these structures are able to take this role with methodological and technical capacity, given the main national program in effect.

## CONCLUSION

The mental health care model advocated by the Brazilian Ministry of Health has been slowly advancing in several aspects and creating urgent demands for its consolidation. The model has assumed, since 2010, the area of crack cocaine and other drugs as a priority focus. In all documents, that line of action is emphasized, being always present in the mental health policy. The PCPV assumes a role of great importance at this moment of the mental health policy, determining some anachronism in relation to the model and the funding. We are not only contrasting the focus of the program, which, as we have discussed, has the admission as expected result, but we are also thinking about the financial impact within a reality of chronic underfunding of the health, and mental health as a technical area.

The increase in the total budget of the health area would entail in the densification of services and offers; however, without a change in the organizational process, this growth would lose power because of a fragmented organizational and irrational logic, as we have analyzed. The debate about governance and organization of mental health services, in this perspective, is an agenda that must enter the program of mental health and social control policy makers. The funding of the area needs to expand its relative percentage, since demand tends to increase, given the undergoing demographic transition.

The assessment has shown to be a subject that is not present in the practices of the bureaucratic field. There is a gap between the studies on the evaluative-academic field and their incorporation as what could move the model forward. In this current time of consolidation of the psychosocial care model, the creation of a Psychosocial Care Index would not be excessive, as it could, by using indicators, capture the main components of the current policy and its objectives to support the efforts of workers and managers, give greater transparency to users and society, and identify weak points that could be improved.

It is important to relate the analyses of this study to the dimensions of the institutional and professional practices, as the reproduction of different models can be sustained on the particularity of the micropolitical relations. The models will never be pure, but they can be a synthesis between what is proposed and induced by the official public policies (which we have just analyzed) and what is built in the daily life of the service-professional-user relationship.

## Footnotes

a Brasil. Lei nº 10.2016, de 6 de abril de 2001. Dispõe sobre a proteção e os direitos das pessoas portadoras de transtornos mentais e redireciona o modelo assistencial em saúde mental. Brasília (DF); 2001 [cited 2017 Jan 13]. Available from: http://www.planalto.gov.br/ccivil_03/leis/leis_2001/l10216.htm4

b Saúde Mental em Dados. Brasília (DF): Ministério da Saúde; 1995;10(12). Available from: http://www.mhinnovation.net/sites/default/files/downloads/innovation/reports/Report_12-edicao-do-Saude-Mental-em-Dados.pdf

c Brasil. Decreto nº 7.508, de 28 de junho de 2011. Regulamenta a Lei nº.8.080, de 19 de setembro de 1990, para dispor sobre a organização do Sistema Único de Saúde - SUS, o planejamento da saúde, a assistência à saúde e a articulação interfederativa, e dá outras providências. Capítulo 2, Seção I – Das Regiões de Saúde [cited 2014 Jun 23]. Available from: http://www.planalto.gov.br/ccivil_03/_ato2011-2014/2011/decreto/D7508.htm

d Ministério da Saúde (BR). Portaria nº 3.088, de 23 de dezembro de 2011. Institui a Rede de Atenção Psicossocial para pessoas com sofrimento ou transtorno mental e com necessidades decorrentes do uso de crack, álcool e outras drogas, no âmbito do Sistema Único de Saúde (SUS) [cited 2014 Jun 23]. Available from: http://bvsms.saude.gov.br/bvs/saudelegis/gm/2011/prt3088_23_12_2011_rep.html

e Brasil. Decreto nº 7.179, de 20 de maio de 2010. Institui o Plano Integrado de Enfrentamento ao Crack e outras Drogas, cria o seu Comitê Gestor, e dá outras providências. [cited 2014 Jun 23]. Available from: http://www.planalto.gov.br/ccivil_03/_Ato2007-2010/2010/Decreto/D7179.htm

f Ministério da Saúde (BR), Conselho Nacional de Saúde. Resolução Nº 448, de 6 de outubro de 2011. [cited 2014 Jun 23]. Available from: http://conselho.saude.gov.br/resolucoes/2011/Reso448.doc

g Ministério da Saúde (BR). Portaria nº 4.279, de 30 de dezembro de 2010. Estabelece diretrizes para a organização da Rede de Atenção à Saúde no âmbito do Sistema Único de Saúde (SUS). [cited 2014 Jun 23]. Available from: http://bvsms.saude.gov.br/bvs/saudelegis/gm/2010/prt4279_30_12_2010.html

h World Health Organization, Regional Office for Europe. The European Mental Health Action Plan. Copenhagen; 2013 [cited 2014 Jun 21]. Available from: http://www.euro.who.int/__data/assets/pdf_file/0004/194107/63wd11e_MentalHealth-3.pdf

i Relatório final da IV Conferência Nacional de Saúde Mental -Intersetorial; 17 jun-1 jul 2010; Brasília (DF). Brasília (DF): Ministério da Saúde, Conselho Nacional de Saúde; 2010.

j Ministério da Saúde (BR). Portaria nº 130, de 26 de janeiro de 2012. Redefine o Centro de Atenção Psicossocial de Álcool e outras Drogas 24h (CAPS AD III) e os respectivos incentivos financeiros. [cited 2014 Jun 23]. Available from: http://bvsms.saude.gov.br/bvs/saudelegis/gm/2012/prt0130_26_01_2012.html

k Ministério da Saúde (BR). Portaria nº 131, de 26 de janeiro de 2012. Institui incentivo financeiro de custeio destinado aos Estados, Municípios e ao Distrito Federal para apoio ao custeio de Serviços de Atenção em Regime Residencial, incluídas as Comunidades Terapêuticas, voltados para pessoas com necessidades decorrentes do uso de álcool, crack e outras drogas, no âmbito da Rede de Atenção Psicossocial [cited 2014 Jun 23]. Available from: http://bvsms.saude.gov.br/bvs/saudelegis/gm/2012/prt0131_26_01_2012.html
